# Supplementary material for: Machine intelligence identifies soluble TNFa as a therapeutic target for spinal cord injury
Source: Sci Rep. 2021 Feb 9;11:3442. doi: 10.1038/s41598-021-82951-5 (PMC7873211; doi:10.1038/s41598-021-82951-5)
Supplement: Supplementary file 1 — Supplementary Information [file 41598_2021_82951_MOESM1_ESM.docx]

Machine intelligence identifies soluble TNFa as a therapeutic target for spinal cord injury

JR Huie^1^*, AR Ferguson^1,2^*, N Kyritsis^1^ , J Z Pan^3^, K-A Irvine^4^, JL Nielson^5,6^ , PG Schupp^7^, MC Oldham^7^ , JC Gensel^8^, A Lin^1^, MR Segal^9^, RR Ratan^10^, JC Bresnahan^1^, MS Beattie^1^.

**Supplemental Table 1. Anti-inflammatory drug studies.** Parameters of drug type and dose for each drug study that was included in the initial data-driven analysis of multivariate treatment effects (N=159 rats).

**Supplemental Figure 1.**


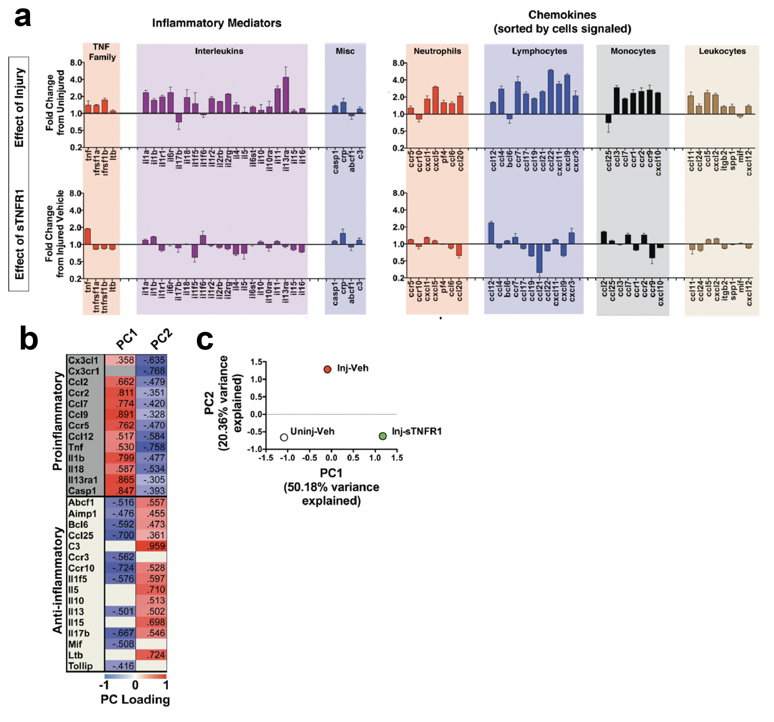


**Supplemental Figure 1. Effect of sTNFR1 on inflammatory gene panel.** A multiplexed PCR panel of 84 inflammatory genes was used to assess the broad effect of sTNFR1 treatment on neuroinflammation after SCI. sTNFR1 was delivered i.t. 90 minutes after injury, and spinal cord was harvested for PCR 3 hours after injury. **a**, Overall expression levels were increased in the vehicle-treated SCI group, as expressed by fold-change relative to uninjured sham control subjects (“Effect of Injury”). sTNFR1 treatment either dampened or reversed this neuroinflammatory response in the majority of genes tested. (“Effect of sTNFR1”). **b**, Principal components analysis of all gene expression values across all testing groups revealed two principal components that together accounted for 71% of the variance. PC1 was characterized by high positive loadings in classically pro-inflammatory genes, and PC2 was most strongly driven by anti-inflammatory genes. **c,** biplot of the three experimental groups on the PC1 and PC2 axes illustrates the 2 dimensional syndromic space occupied by each experimental condition. sTNFR1 treatment (Inj-sTNFR1) produces an inflammatory profile that is distinct from both injury alone (Inj-Veh) and uninjured controls (Uninj-Veh).

**Supplemental Table 2.**

**Supplementary Table 2.** 295 genes were significantly up- or downregulated after sTNFR1 injection. Terms from Gene Ontology analysis indicate that 95 of 295 (32.2%) differentially expressed genes were related to inflammatory processes. Columns 1 and 2 indicate the specific Gene Ontology term numbers and descriptions. P-value indicates nominal significance value for each gene, while FDR q-value reflects the adjusted value accounting for false discovery rate. Enrichment score indicates the degree to which the genes are overrepresented at the top or bottom of the entire ranked list of genes.
